# Heightened sensitivity to high-calorie foods in children at risk for obesity: insights from behavior, neuroimaging, and genetics

**DOI:** 10.1007/s11682-023-00773-7

**Published:** 2023-05-05

**Authors:** Kristina M. Rapuano, Link Tejavibulya, Eda Naz Dinc, Anfei Li, Haley Davis, Rachel Korn, Rudolph L. Leibel, B. Timothy Walsh, Lisa Ranzenhofer, Michael Rosenbaum, B. J. Casey, Laurel Mayer

**Affiliations:** 1 Department of Psychology, Yale University, 2 Hillhouse Ave, New Haven, CT 06511 USA; 2 Interdepartmental Neuroscience Program, Yale University, New Haven, CT USA; 3 Department of Psychiatry, Weill Cornell Medical College, New York, NY USA; 4 Department of Psychiatry, Columbia University Medical College, New York, NY USA; 5 Department of Pediatrics, Columbia University Medical College, New York, NY USA

**Keywords:** fMRI, Go/no-go, Food cues, Salience network, Obesity risk

## Abstract

Pediatric obesity is a major public health concern. Genetic susceptibility and increased availability of energy-dense food are known risk factors for obesity. However, the extent to which these factors jointly bias behavior and neural circuitry towards increased adiposity in children remains unclear. While undergoing fMRI, 108 children (ages 5-11y) performed a food-specific go/no-go task. Participants were instructed to either respond (“go”) or inhibit responding (“no-go”) to images of food or toys. Half of the runs depicted high-calorie foods (e.g., pizza) whereas the other half depicted low-calorie foods (e.g., salad). Children were also genotyped for a DNA polymorphism associated with energy intake and obesity (*FTO* rs9939609) to examine the influence of obesity risk on behavioral and brain responses to food. Participants demonstrated differences in behavioral sensitivity to high- and low-calorie food images depending on task demands. Participants were slower but more accurate at detecting high- (relative to low-) calorie foods when responding to a neutral stimulus (i.e., toys) and worse at detecting toys when responding to high-calorie foods. Inhibition failures were accompanied by salience network activity (anterior insula, dorsal anterior cingulate cortex), which was driven by false alarms to food images. Children at a greater genetic risk for obesity (dose-dependent model of the *FTO* genotype) demonstrated pronounced brain and behavioral relationships such that genetic risk was associated with heightened sensitivity to high-calorie food images and increased anterior insula activity. These findings suggest that high-calorie foods may be particularly salient to children at risk for developing eating habits that promote obesity.

**Supplementary Information:**

The online version contains supplementary material available at 10.1007/s11682-023-00773-7.

## Introduction

Over 35% of youth and 73% of adults in the United States are considered overweight or obese (Fryar et al., 2018a, 2018b). Obesity increases risk for hypertension, type 2 diabetes, coronary heart disease, stroke, cancer (National Heart, Lung, and Blood Institute 1998), and mortality (Flegal et al., 2005). Childhood overweight and obesity are strong predictors of obesity later in life (Simmonds et al., 2016; Wang et al., 2008), with consumption of calorie-dense and ultraprocessed food (Chang et al., 2021; Neri et al., 2022; Sahoo et al., 2015) and genetics (Elks et al., 2012; Farooqi & O’Rahilly, 2000) among the greatest risk factors for the development of obesity. Although food-related impulsivity and lack of inhibitory control are associated with unhealthy eating and obesity (Thamotharan et al., 2013), the behavioral and neurobiological mechanisms underlying food-related impulsivity and their interactions with genetic risk for obesity remain poorly understood.

Response inhibition is important for suppressing approach behaviors that may become impulsive (e.g., unhealthy eating) or lead to negative health outcomes (e.g., obesity) (Casey, 2015; Inzlicht et al., 2021; Moffitt et al., 2011). The motivational salience of cues that signal reward is determined by behavioral relevance or subjective value (Berridge, 2012; Simmons et al., 2013a, 2013b) and can impact behavioral and neural processes involved in impulse control (Higgs et al., 2015; Lopez et al., 2017; Wagner et al., 2013). For example, heightened impulsivity to palatable food images has been observed in healthy-weight individuals (Deux et al., 2017; Teslovich et al., 2014) and in overweight/obese individuals (Giel et al., 2017; Nederkoorn et al., 2012; Schag et al., 2013). Understanding impulse control is particularly important in individuals who are learning and establishing behaviors that may become habitual later in life. Response inhibition continues to emerge through adolescence (Casey et al., 2011; Tamm et al., 2002) and has been suggested to primarily engage the cingulo-opercular network in youth (Roe et al., 2021). The cingulo-opercular network has been functionally referred to as the “salience network” (Seeley et al., 2007; Uddin et al., 2019), with the anterior insula in particular considered to be involved in the detection of behaviorally-relevant or subjectively salient stimuli (Uddin, 2015).

In addition to cognitive and affective influences on impulsivity, genetic factors can further bias eating behavior and susceptibility to unhealthy food choices. The fat-mass and obesity-associated (*FTO*) rs9939609 polymorphism is highly associated with obesity-related metrics across the lifespan (Frayling et al., 2007) and facilitates weight gain by increasing energy-dense food intake (Cecil et al., 2008; Church et al., 2010; Gilbert-Diamond et al., 2017; Haupt et al., 2009; Speakman et al., 2008), including in children without obesity (Ranzenhofer et al., 2019). Using a dose-dependent model of this polymorphism as an indicator of obesity risk, heightened neural responses to food cues in genetically at-risk children has been observed (Rapuano et al., 2017). Thus, children with more copies of the *FTO* rs9939609 risk allele (A) may be at a greater risk for developing obesity due to enhanced reactivity to food cues—potentially leading to increased consumption of energy-dense foods.

Here we examine variability in behavioral and brain responses to food cues during an in-scanner impulse control task as a function of task demands (intra-subject variability) and genetic risk for obesity (inter-subject variability). More specifically, we instruct 5–11-year-old children to either respond (“go)—or inhibit responding (“no-go”)—to food images, allowing us to test competing hypotheses about response inhibition to food images compared to neutral toy images (i.e., whether children exhibit increased inhibition failure to foods versus increased sensitivity/performance due to enhanced salience of food cues). In addition, we separately present children with either high- or low-calorie food images to further explore the motivational salience of palatable foods during an impulse control task. Finally, we test the hypothesis that genetic risk for obesity increases the salience of high-calorie food images.

## Methods

### Participants

Participants were a community sample of 108 children (5–11 years; mean [s.d.] age: 8.88 [1.3] years; 57 females) (Table 1). Recruitment procedures are described elsewhere (Ranzenhofer et al., 2019). Exclusion criteria included MRI contraindications, medical conditions related to diet (e.g., diabetes, eating disorders), and medications that can impact food intake. Height and weight were used to estimate body mass index *z*-scores (BMI-*z*) in accordance with CDC growth charts. Ten participants were unable to complete the scan (e.g., unable to acclimate to scanner; technical difficulties). Imaging data from the remaining participants were visually inspected for artifacts and three participants were excluded due to excessive motion, resulting in a neuroimaging sample of 95 participants (Table S1). Participants provided assent and parents/guardians provided consent in accordance with the New York State Psychiatric Institute/Columbia University IRB-approved guidelines.

**Table 1:** Demographics for all participants. Values represent mean (s.d.) unless indicated otherwise. *P*-values represent statistical significance of differences across genotypes (i.e., Hardy–Weinberg equilibrium test for the distribution of genotypes in the current sample; one-way analysis of variance [ANOVA] tests for age, BMI-*z*, and biological sex)

	All subjects	AA	AT	TT	*p*-value
*n*	108	26	51	30	0.90
Age	8.88 (1.35)	8.48 (1.49)	9.09 (1.29)	8.92 (1.30)	0.18
BMI-*z*	0.31 (0.94)	0.52 (1.03)	0.24 (0.98)	0.27 (0.78)	0.45
Sex (%F)	52.78	48.15	55.77	58.06	0.70
% White	43.52	61.54	39.22	33.33	–
% Black	20.37	7.69	25.49	13.33	–
% Asian	7.41	0.00	5.88	16.67	–
% Hispanic	47.22	26.92	49.02	63.33	–

### FTO rs993960 genotyping

DNA was prepared and extracted from saliva via DNA Genotek™ kits for each participant prior to the scan session. Children were genotyped for the *FTO* rs993960 SNP using pyrosequencing (PSQ96 Biotage, LLC). PCR reactions consisted of 6-pmol of each of the forward and reverse primer, 0.75-U GoTaq, 1xGoTaq buffer, 0.2-mM dNTP's and 50-ng of genomic DNA in a 30-μλ reaction volume for 35 cycles at an annealing temperature of 50 °C. Insufficient specimen quantity led to an unknown genotype for one participant. The distribution of genotypes across the remaining 107 participants (26 AA; 51 AT; 30 TT) was consistent with the Hardy–Weinberg equilibrium (χ2 = 0.22; p-value = 0.90), and did not differ by age, sex, or BMI-*z* (p’s > 0.3) (Table 1; Figure S1).

### Study design

**Fig. 1:**
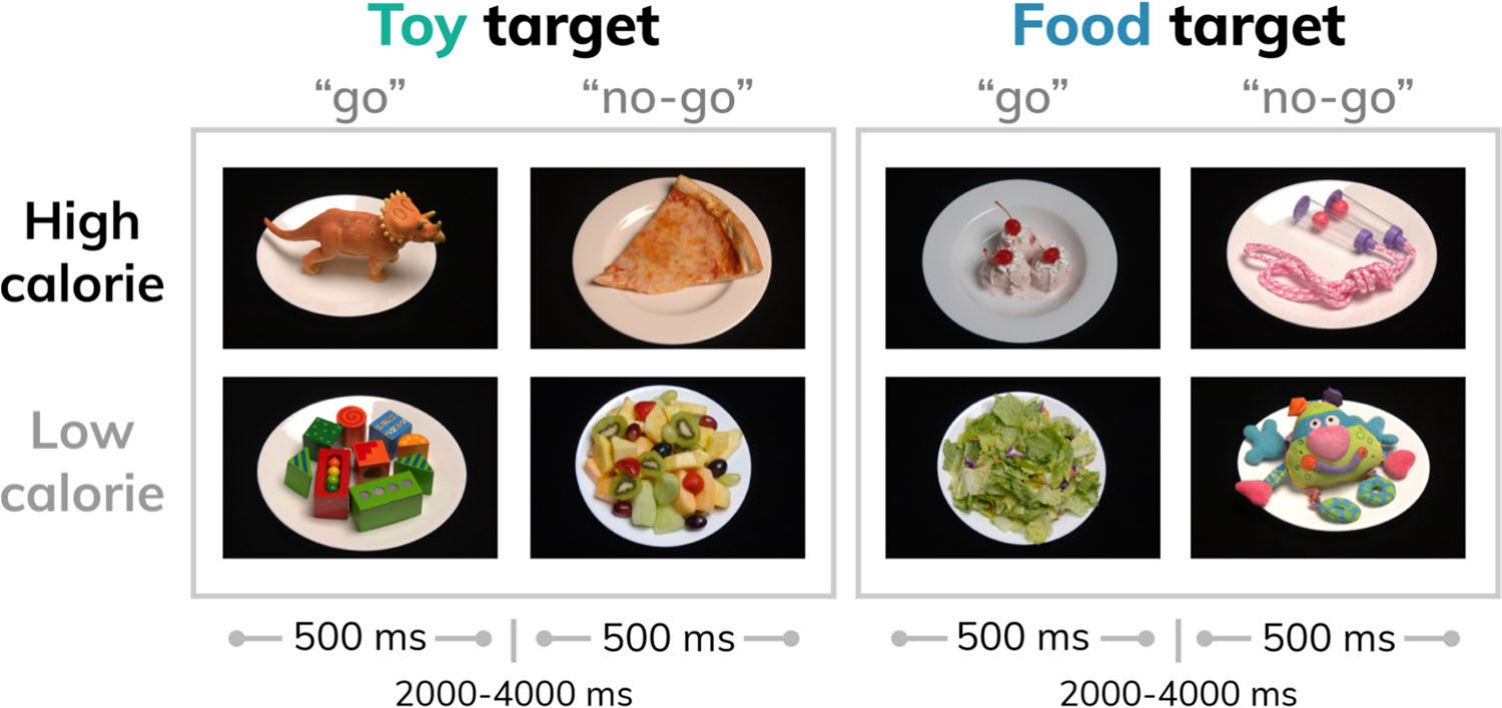
Study design schematic. Food (high- or low-calorie) and toy images were presented during four task runs (run order counterbalanced across participants). Participants were instructed to “go” to food images and or “no-go” to toys for two runs, and to “go” to toy images and or “no-go” to food for two runs

Participants performed a food-specific go/no-go task during functional magnetic resonance imaging (fMRI). Stimuli were comprised of 30 images of high- (n = 8; e.g. pizza, ice cream) and low-calorie (n = 7; salad; fruit) food images and images of toys (n = 15). Additional details about the stimulus set are reported elsewhere (Teslovich et al., 2014). Participants were instructed to respond to toy images and inhibit responding to food images during half of the runs, and to respond to food images and inhibit responding to toys during the other half (Fig. 1). Participants completed four runs counterbalanced for order of presentation with high-calorie foods depicted in half of the runs and low-calorie foods depicted in the other half, such that each participant completed trials where they responded (“go”) to high- and low-calorie foods and inhibited responses (“no-go”) to high- and low-calorie foods. For example, a participant would be presented with images of pizza and ice cream during half of the runs, and would either respond or inhibit responding to these images depending on the task instructions for a given run.

For each run, targets and distractors comprised 70% and 30% of the trials, respectively. To control for satiety levels, participants were instructed not to eat or drink (other than water) past 10PM the night prior to the scan and were provided with a standardized breakfast (adjusted for estimated energy expenditure based on age, sex, and weight) the morning of the scan.

### Image acquisition

Scans were collected on a 3 T GE Discovery MR750 using a 32-channel headcoil. Structural images were acquired using a T1-weighted 3D BRAVO (BRAin VOlume imaging) sequence at a resolution of 1 × 1 × 1 mm^3^ (dimensions: 256 × 256 × 176 slices interleaved; FOV: 25.6; inversion time: 450 ms; flip angle: 12°). Functional scans were acquired using a T2*-weighted echo-planar imaging sequence with a spatial resolution of 3.0 mm^3^ (TR: 2500 ms; TE: 30 ms; flip angle: 72°; FOV: 19.2; dimensions: 64 × 64 × 34 slices interleaved).

### Behavioral analysis

For each participant, behavioral sensitivity (*d’*) during each task condition was calculated by subtracting normed false alarm rates from normed hit rates using the percentile point function (ppf) in *scipy* (Virtanen et al., 2020). In the context of the current paradigm, this measure was calculated by comparing the proportion of commission errors on “no-go” trials relative to the proportion of correct hits on “go” trials (i.e., responding to food images and inhibiting responses to toy images for half the runs, and vice versa for the other half). Reaction times were averaged for each task condition. Response times less than 100 ms were considered subthreshold for perceptual awareness and ignored. All statistical analyses described in the current manuscript utilized the *lme4* package (Bates et al., 2015) in R (R Core Team, 2020). Differences in *d ‘* and reaction times as a function of task demands and calorie load were estimated using linear mixed-effects models. Models included age, biological sex, self-reported race and ethnicity, BMI-*z*, and run order as covariates as well as random intercepts for subject. Similar models were performed to test for behavioral differences by genetic risk for obesity, with the inclusion of interaction terms for *FTO* genotype. Genetic risk based on the *FTO* rs993960 polymorphism was modeled linearly, following an additive model of risk. Although BMI-*z* did not significantly differ across genotype in the current sample, BMI-*z* was included as a covariate to account for differences that have been reported in previous work (Frayling et al., 2007) and that emerge over the course of development (Haworth et al., 2008).

### fMRI preprocessing and first-level analyses

Neuroimaging data for 95 participants were preprocessed using *fmriprep* (Esteban et al., 2019), including skull stripping, spatial realignment, and boundary-based nonlinear registration to MNI-coordinate space. Resulting images were scaled by the mean of each run and submitted to a first-level GLM using AFNI to estimate voxel-wise responses during the Go/No-go task. To account for potential sources of noise, nuisance regressors included the 24-motion parameter Volterra expansion, anatomical component-based noise correction, framewise displacement, and global signal outliers (> 3 s.d.). Events were modeled according to each participant’s trial-wise reaction time to isolate cognitive processes involved in the Go/No-go task.

### fMRI group-level analysis

A group-level analysis was run comparing activity during commission errors (false alarms) to correct hits, thereby controlling for motor responses and isolating a whole-brain contrast specific to impulsivity. To identify significant clusters of activity across subjects, AFNI’s Equitable Thresholding and Clustering (Cox, 2019) was used with variable spatial blurring inputs, which reduces false positive rates inherent to traditional parametric approaches that rely on spatial autocorrelation of fMRI noise (Eklund et al., 2016). Resulting clusters were further interrogated via region of interest (ROI) analysis.

Mean activity during false alarm trials was extracted for each ROI and each participant. Events were separated into high- and low-calorie false alarms (i.e., incorrect “no-go” when food is presented as a distractor), as well as toy false alarms during high- and low-calorie target blocks (i.e., incorrect “no-go” to toys when food is presented as the target). To test for differences between task conditions (i.e., inhibit responses to food versus toys) and calorie load, resulting estimates were entered into a linear mixed-effects model with random intercepts for ROI nested within subject. Covariates included age, sex, race and ethnicity, BMI-*z*, and run order. A similar model was used to test for differences in insula activity by genetic risk for obesity, with the inclusion of an interaction term for genotype by calorie load. Consistent with the behavioral analysis, genetic risk was modeled linearly in accordance with an additive model of the *FTO* rs993960 polymorphism.

## Results

### Behavioral sensitivity to high- and low-calorie food images is influenced by task demands

**Fig. 2:**
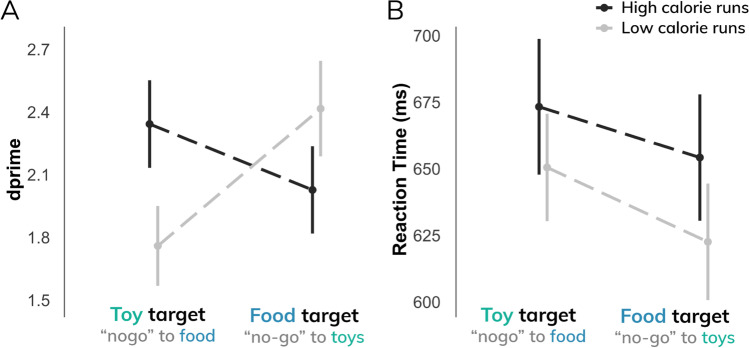
**A**) Behavioral sensitivity (*d’*) to food images relative to toy images differs by task demands (i.e., inhibiting responses to food images [distractor] or responding to food [target]) and stimulus type (i.e., high-calorie; low-calorie images). **B**) Response times to target images were significantly slower during high-calorie runs compared to low-calorie runs

Participants demonstrated greater behavioral sensitivity (*d’*) to high-calorie images (ME of calorie load: β = 0.57; t = 3.69; p < 0.001), particularly when food images were distractors (ME of target type: β = 0.44; t = 2.85; p < 0.005; interaction: β = 1.01; t = 4.60; p < 0.001), suggesting that high-calorie images may be more salient than low-calorie images (Fig. 2A). This effect appeared to be driven by differences in false alarm rates; that is, participants made fewer commission errors to high-calorie images (β = –0.10; t = 4.56; p < 0.001), particularly when high-calorie foods were distractors (interaction: β = 0.21; t = 6.51; p < 0.001). Independent of calorie type, participants made fewer commission errors when food items, rather than toys, were presented as the distractor (ME of target type: β = –0.14; t = 6.19; p < 0.001) (Figure S2A). There were no significant differences in hit rates between calorie load (p > 0.5) or target type (p > 0.3) (Figure S2B). These findings suggest that behavioral performance differences (*d’*) were attributable to commission errors rather than an overall ability to discriminate food from toys.

In addition, participants responded more slowly to targets during high-calorie runs relative to low-calorie runs (β = 26.36; t = 2.73; p < 0.01) and were marginally slower in responding to target images when food was the distractor (β = 15.82; t = 1.63; p = 0.1) (Fig. 2B).

### Brain activity during commission errors differs by task demands

**Fig. 3:**
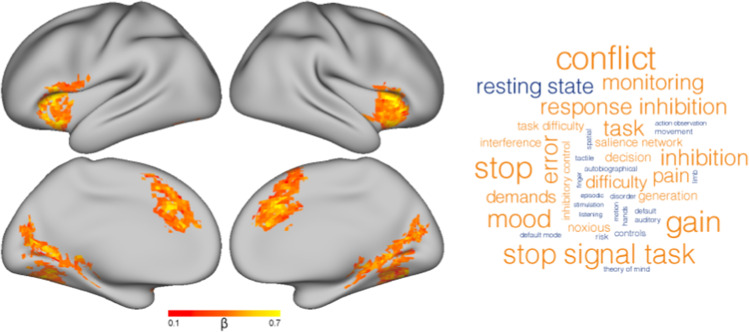
Cluster-corrected whole-brain contrast representing activity during False Alarms relative to Hits across all stimulus types (left). Wordcloud representing the top 20 positive (orange) and negative (blue) terms associated with the activation map (right)

Across all stimulus categories, commission errors (relative to hits) engaged bilateral anterior insula (aIns), dorsal anterior cingulate cortex (dACC) extending into supplemental motor cortex, and cuneus extending along the lingual gyrus (Fig. 3). Using Neurosynth’s Image Decoder (Yarkoni et al., 2011), meta-analytic terms associated with this distributed pattern of activity were identified and visualized according to the strength of their associations. Terms that were strongly correlated with this contrast included “conflict”, “stop”, and “error”, whereas “resting state” was strongly inversely associated.

**Fig. 4:**
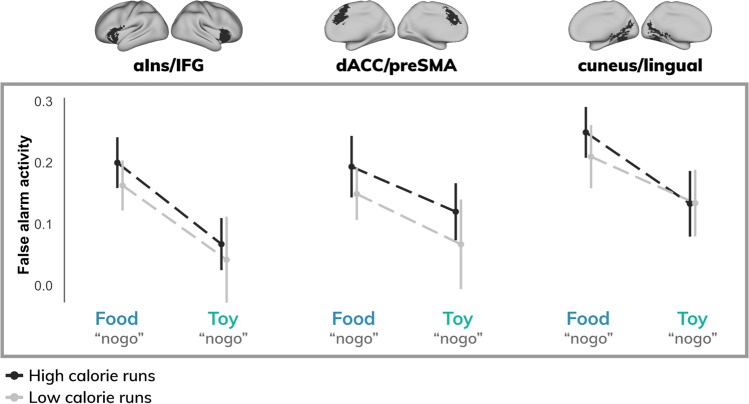
Activity for each region of interest stratified by task demands and calorie load. Across all participants, food false alarms elicited stronger activity (top) relative to toy false alarms (bottom). No significant differences were observed between high- and low-calorie runs. [aIns = anterior insula; IFG = inferior frontal gyrus; dACC = dorsal anterior cingulate cortex; preSMA = pre-supplementary motor area]

To examine potential differences in activity across stimulus types and task conditions, regions were further interrogated via ROI analysis. Across ROIs, a main effect of distractor condition was observed (β = 0.11; t = 3.99; p < 0.001), such that false alarm activity was enhanced when participants incorrectly responded to food (relative to toy) images (Fig. 4).

### Behavioral and brain sensitivity to food images differs by genetic risk for obesity

**Fig. 5:**
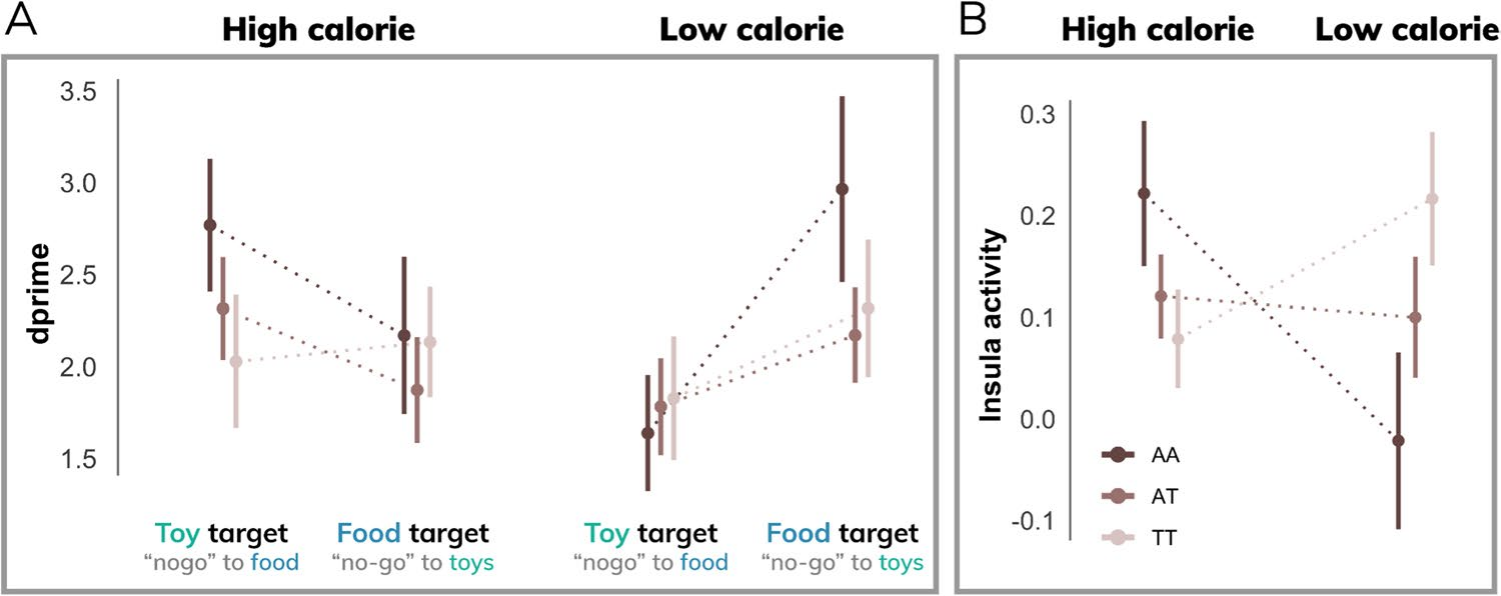
Behavior and brain activity stratified by *FTO* genotype. **A**) Behavioral performance (*d’*) differs by genetic risk for obesity based on an additive model of the *FTO* rs9939609 polymorphism. Compared to low-risk individuals (TT), participants with a higher genetic risk for obesity (one or more A alleles) demonstrated greater behavioral performance in the high-calorie (relative to low-calorie) condition. **B**) Higher-risk individuals showed increased anterior insula activity during high-calorie (relative to low-calorie) runs compared to lower-risk individuals

Genetic risk for obesity, measured by an additive model of the *FTO* rs9939609 genotype, demonstrated a significant interaction with discriminability (*d’*) for high- versus low-calorie food images (interaction: β = 0.58; t = 2.20; p < 0.05), particularly when high-calorie images were the distractor (3-way interaction: β = 0.87; t = 2.36; p < 0.02) (Fig. 5A). In other words, children at a greater genetic risk for obesity exhibited enhanced behavioral sensitivity to high-calorie distractor images. This effect was driven by a significant difference in false alarm rates (3-way interaction: β = 0.12; t = 2.01; p < 0.05) such that at-risk children made fewer false alarms to high-calorie distractor images (Figure S3A). Higher-risk participants were also slower to respond to food target images relative to toys (β = 39.29; t = 2.12; p < 0.05) and slower to respond to targets during high-calorie runs (β = 34.79; t = 1.98; p < 0.05) (Figure S3B). Hit rates did not differ across genotypes for calorie load (p > 0.1) or target type (p > 0.8). Results were consistent without accounting for BMI as a covariate (see supplemental materials).

Moreover, error-related activity in the anterior insula exhibited an interaction between calorie load and genetic risk for obesity (β = 0.16; t = 3.02; p < 0.005) (Fig. 5B). Specifically, individuals with a higher risk of obesity demonstrated greater false alarm activity during high-calorie runs. Results do not change if behavioral performance (*d’*) is included as a covariate.

## Discussion

The current study leverages a food-specific go/no-go task to explore variability in behavioral and brain responses to food cues and their interactions with genetic risk for unhealthy eating behaviors and obesity. We observed task-dependent differences in behavioral sensitivity to food images, such that discriminability was influenced by task demands (i.e., respond versus inhibit response to food) and stimulus content (i.e., calorie load). More specifically, participants were more effective at detecting and inhibiting responses to high- (relative to low-) calorie foods in the context of a neutral stimulus (toys). By contrast, participants demonstrated decreased inhibitory control when responding to high- (relative to low-) calorie food images. There were no differences in hit rate by target type, suggesting that these effects were specific to response inhibition failure and could not be explained by difficulty detecting food versus toys. The interaction between calorie load (high versus low) and target type may, however, be explained by differences in attentional bias and cognitive control. Padmala and Pessoa (2011) found that motivation enhances attentional filtering and reduces task-irrelevant information, suggesting that the salience of a cue can minimize conflict and improve performance. For example, problem gamblers demonstrate *improved* response inhibition to gambling-related cues (van Holst et al., 2012). Slower reaction times during neutral “go” trials observed in the current study may indicate proactive inhibition (Aron, 2011; Criaud et al., 2012), or a motor slowing in preparation for inhibiting a response to a salient stimulus (i.e., high-calorie food). This hypothesis aligns with the enhanced inhibition to high-calorie foods observed here, and may also indicate a lack of proactive inhibition when responding to food images and inhibiting responses to toys.

This hypothesis is further supported by heightened salience network activity during false alarms to food (relative to toy) images. In addition to regions involved in motor preparation (i.e., pre-SMA) and visual attention (i.e., cuneus), inhibitory failures to food robustly engaged the cingulo-opercular network (i.e., aIns; dACC). The cingulo-opercular network has been implicated in executive control (Dosenbach et al., 2007, 2008), and the anterior insula in particular is considered to represent subjective salience or behaviorally-relevant stimuli (Uddin, 2015; Uddin et al., 2019). Motivationally salient stimuli upregulate or engage control-related networks that bias attention, leading to more efficient stimulus processing during conflict (Padmala & Pessoa, 2011). This finding is in line with recent work suggesting that salience processing increases proactive inhibitory activity in the insula/operculum (Gavazzi et al., 2021), which may indicate an effortful attempt of this network to (unsuccessfully) inhibit a motor response (Ghahremani et al., 2015). Taken together with the behavioral differences observed here, these findings suggest that food cues are more salient compared to toys and may contribute to attentional bias toward high-calorie foods or enhanced impulsivity to food more generally.

In addition to influences of task demands and stimulus type, we observed differences in behavioral and brain responses associated with genetic risk for hyperphagic obesity based on an additive model of the *FTO* rs9939609 polymorphism. Participants at a greater genetic risk for developing obesity demonstrated pronounced relationships in both behavioral performance and anterior insula activity during commission errors. Specifically, higher-risk participants (based on a gene dosage effect) showed a greater difference in behavioral sensitivity to high- and low-calorie foods as a function of task demands, as well as a greater difference in salience-related brain activity during impulsive actions to high- (versus low-) calorie foods. Previous work using computational reinforcement learning has shown improved learning rates in individuals with obesity (Kube et al., 2020), which may extend to at-risk individuals prior to obesity onset via *FTO* rs9939609 regulation of dopamine-dependent reward learning (Sevgi et al., 2015). Taken together, these findings suggest that at-risk children may demonstrate enhanced reward learning through increased salience of behaviorally relevant food cues.

These findings should be interpreted with consideration of potential limitations. First, the design of the current study did not include runs where no food stimuli were presented. Thus, the current study is limited in its ability to make inferences beyond food-specific associations. Second, the sample included in the current study may be underpowered to detect meaningful differences associated with a single candidate gene. Replication samples are needed to validate the findings reported here, including controlling for other genes affecting ingestive behaviors. Finally, interoceptive or homeostatic states (e.g., hunger; blood glucose concentrations) have been shown to modulate insula responses to food cues (Simmons et al., 2013a, 2013b), including during a go/no-go task (He et al., 2019), and to influence the representation of subjective salience (Uddin, 2015). Although we sought to control for hunger levels prior to the scan, future work should consider the impact of individual variability in interoceptive and homeostatic signaling on insula responses to food cues.

Unhealthy eating habits established early in life are more difficult to override later in life (Birch et al., 2007; Craigie et al., 2011) and may give rise to neurobiological changes that can perpetuate further unhealthy eating (Décarie-Spain et al., 2018; Rapuano et al., 2022). Prior work even in healthy, nonobese individuals suggests sensitivity to food cues (relative to nonfood items) develops early and is maintained across development (Teslovich et al., 2014). Greater consideration should be given to the implications of enhanced salience of food cues in at-risk youth and potential avenues to mitigate such responses early in life. For example, calorie labeling has been shown to dampen responses to food cues in brain regions associated with impulse control (Courtney et al., 2019), which may help regulate the motivational salience of high-calorie foods and impulsive food choices in youth.

## Conclusions

The current study suggests that sensitivity to food cues in children is task- and context-dependent, and also interacts with genetic vulnerability to obesity. These findings may inform our understanding of food-based decision-making in youth prior to the development of unhealthy eating habits or obesity—particularly in high-risk individuals.

### Supplementary Information

Below is the link to the electronic supplementary material.

Supplementary file1 (PDF 749 KB)

## Data Availability

Stimuli used in this study can be found at http://fablab.yale.edu/page/assays-tools. Raw fMRI data are available on openneuro.

## Code availability

Processing pipelines and code used for statistical analyses will be provided upon request.
