# Supplementary material for: Heightened sensitivity to high-calorie foods in children at risk for obesity: insights from behavior, neuroimaging, and genetics
Source: Brain Imaging Behav. Author manuscript; Available in PMC 2023 Oct 2. (PMC10543571; doi:10.1007/s11682-023-00773-7)
Supplement: Supplement [file NIHMS1915962-supplement-Supplement.pdf]

Supplemental material

	All subjects	AA	AT	TT	p-value
**n**	95	25	45	25	0.88
**Age**	8.94 (1.38)	8.49 (1.51)	9.11 (1.31)	9.06 (1.30)	0.17
**BMI-*z***	0.30 (0.95)	0.49 (1.04)	0.21 (0.99)	0.27 (0.78)	0.49
**Sex (%F)**	55.79	48.00	60.00	56.00	0.63
**% White**	44.21	64.00	40.00	32.00	–
**% Black**	20.00	20.00	26.67	8.00	–
**% Asian**	22.11	0.00	6.67	20.00	–
**% Hispanic**	5.26	24.00	51.11	68.00	–

Table S1. Demographics for neuroimaging subsample. Values represent mean (s.d.) unless indicated otherwise. P-values represent statistical significance of differences across genotypes (i.e., Hardy–Weinberg equilibrium test for the distribution of genotypes in the current sample; one-way analysis of variance [ANOVA] tests for age, BMI-z, and biological sex).

Supplemental results

BMI was not significantly related to behavioral sensitivity (d') to high-calorie ($p = .71$) nor low-calorie food ($p = .80$), even within only AA children (high-calorie: $p = .23$; low-calorie: $p = .54$). BMI was also unrelated to behavioral sensitivity to food images overall ($p = .98$) nor to toy images ($p = .79$), even within only AA children (food: $p = .36$; toy: .36).

Although there was no significant differences in BMI-z scores by genotype in the current sample, the BMI-z scores for AA children are nearly twice that of AT/TT children (Table 1). As such, we account for this variance by including it as a covariate to hone in on brain/behavioral associations independent of any potential BMI differences. Results were consistent with those reported in the main manuscript, such that genetic risk for obesity, measured by an additive model of the *FTO* rs9939609 genotype, demonstrated a significant interaction with discriminability *(d')* for high- versus low-calorie food images (interaction: $\beta=0.58$; $t=2.21$; $p<.05$), particularly when high-calorie images were the distractor (3-way interaction: $\beta=0.87$; $t=2.36$; $p<.02$). This effect was driven by a significant difference in false alarm rates (3-way interaction: $\beta=0.12$; $t=2.01$; $p<.05$) such that at-risk children made fewer false alarms to high-calorie distractor images. Moreover, error-related activity in the anterior insula exhibited an interaction between calorie load and genetic risk for obesity ($\beta=0.24$; $t=2.71$; $p<.01$).

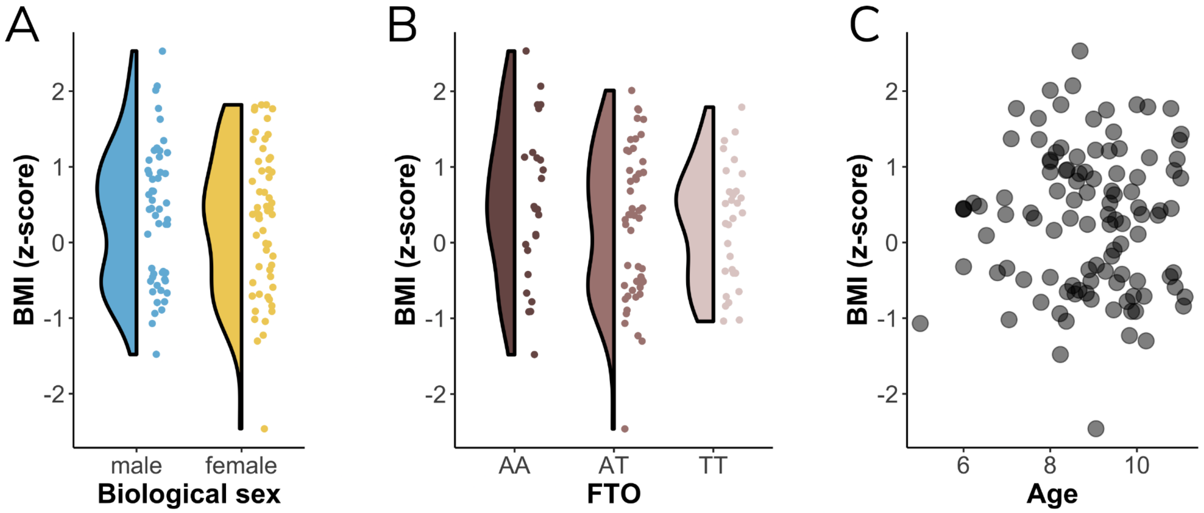

Figure S1. BMI-*z* does not differ by biological sex (A), *FTO* rs9939609 genotype (B), or age (C).

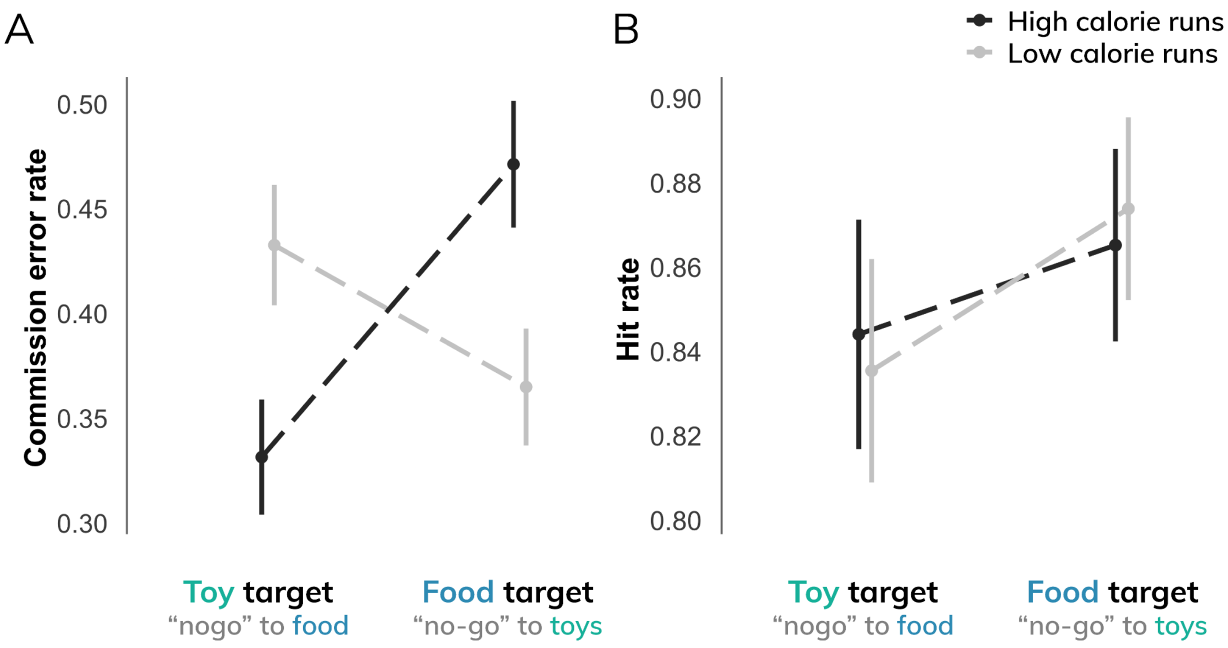

Figure S2. A) Commission error rates differed by task demands (i.e., inhibiting responses to food images [distractor] or responding to food [target]) and stimulus type (i.e., high-calorie; low-calorie images). B) Hit rates showed no differences between target type or calorie load.

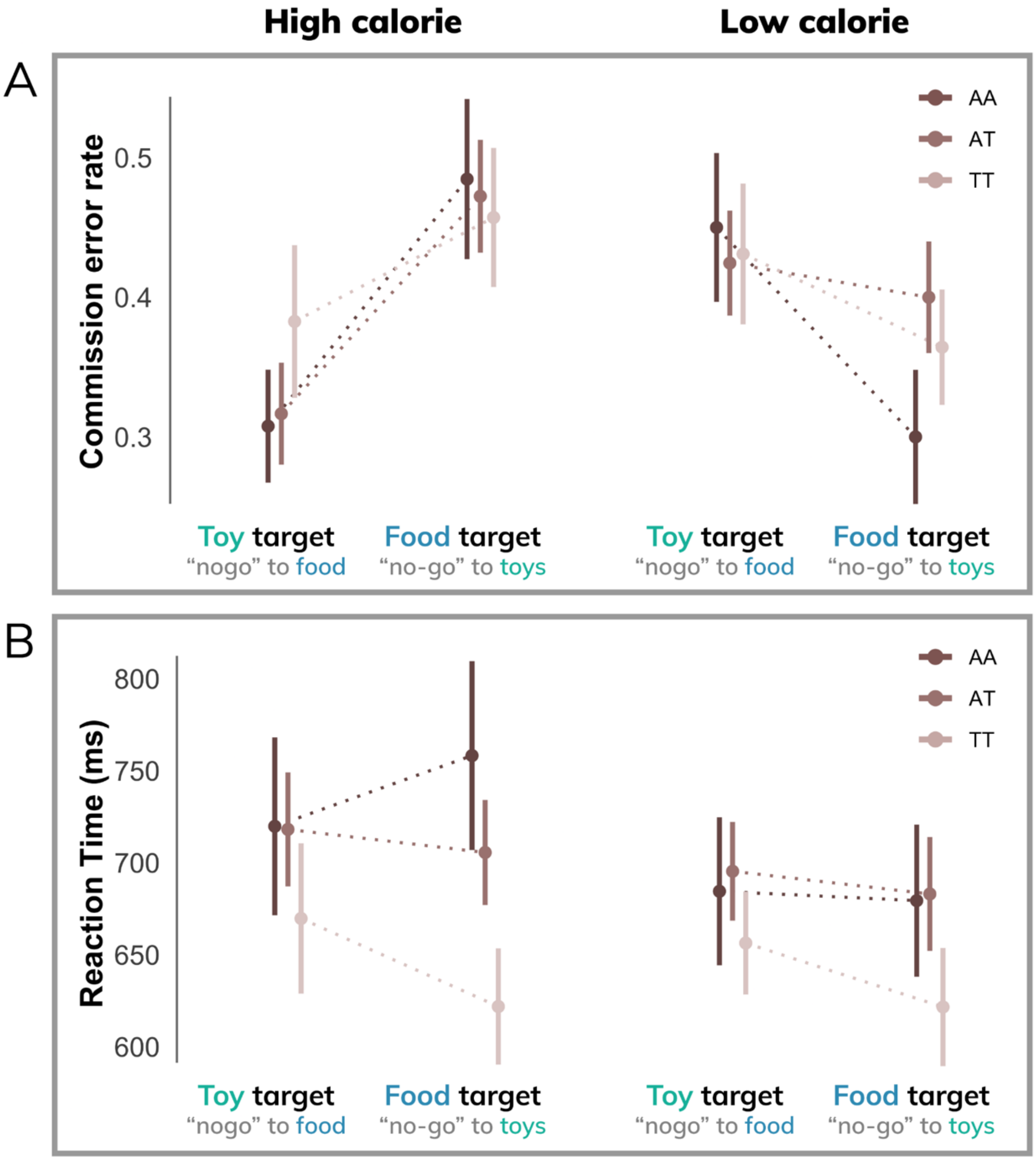

Figure S3. A) False alarm rates differed by genetic risk for obesity based on the FTO rs9939609 polymorphism. Compared to low-risk individuals, participants with a higher genetic risk for obesity made fewer commission errors in the high-calorie (relative to low-calorie) condition. B) Participants at a higher risk for obesity demonstrated slower reaction times to targets during high-calorie runs and when food was the target condition.
